# Role of metabolic characteristics in the co-occurrence of insomnia, Alzheimer’s disease, and Parkinson’s disease: a Mendelian randomization study

**DOI:** 10.3389/fnagi.2024.1436171

**Published:** 2025-01-06

**Authors:** Chengyong Liu, Chi Wang, Jing Jiang, Yuyang Bo, Lixiu Nan, Ying Zhang, Kongxi Zhu, Xiaoqiu Wang, Xinxin Feng, Xiaoyang Lian, Shan Qin

**Affiliations:** ^1^Jiangsu Province Hospital of Chinese Medicine, Affiliated Hospital of Nanjing University of Chinese Medicine, Nanjing, Jiangsu, China; ^2^Shanghai University of Traditional Chinese Medicine, Shanghai, China; ^3^Ningbo Traditional Chinese Medicine Hospital, Ningbo, Zhejiang Province, China

**Keywords:** insomnia, neurodegenerative diseases, Alzheimer’s disease, Parkinson’s disease, Mendelian randomization

## Abstract

**Objective:**

There reportedly exists a significant comorbidity between insomnia and neurodegenerative diseases, such as Alzheimer’s disease (AD) and Parkinson’s disease (PD), indicative of a potential link to serum metabolic dysregulation.

**Method:**

To elucidate shared pathophysiological mechanisms between insomnia and AD/PD, we performed comprehensive two-sample Mendelian randomization (MR) analyses, investigating 1,400 serum metabolic characteristics for their causal relationships with the risks of insomnia, AD, widely defined AD (WDAD), and PD. We employed publicly available genetic data; the primary estimate was determined using inverse-variance weighting, supplemented by weighted median, simple mode, weighted mode, and the MR-PRESSO and MR-Egger methods to evaluate heterogeneity and pleiotropy.

**Results:**

The ratio of N-palmitoyl-sphingosine to N-palmitoyl-sphinganine is linked to higher risks of insomnia (OR = 1.137, 95% CI = 1.015–1.273, *p* = 0.026) and AD (OR = 1.090, 95% CI = 1.005–1.183, *p* = 0.037). The acetylcarnitine to propionylcarnitine ratio is a risk factor for insomnia (OR = 1.190, 95% CI = 1.003–1.370, *p* = 0.016) but has protective effects against AD (OR = 0.868, 95% CI = 0.784–0.961, *p* = 0.006) and WDAD (OR = 0.892, 95% CI = 0.817–0.973, *p* = 0.010). Glutamine conjugate of C7H12O2 levels are associated with reduced risk of insomnia (OR = 0.863, 95% CI = 0.749–0.995, *p* = 0.042) and PD (OR = 0.856, 95% CI = 0.746–0.981, *p* = 0.026).

**Conclusion:**

Our findings highlight the crucial role of serum metabolic characteristics in the comorbidity of insomnia with neurodegenerative diseases, providing valuable insights into prospective therapeutic targets and diagnostic markers.

## 1 Introduction

Despite optimal sleep environments and opportunities, individuals suffering from insomnia tend to express dissatisfaction with both sleep duration and quality, often leading to daytime functional impairments (Morin et al., 2015). The prevalence of insomnia is on the rise owing to rapid societal development, with approximately 10% adults meeting the diagnostic criteria for the condition (Hale et al., 2020). Insomnia poses a significant threat to both physiological and psychological health, substantially increasing the risk of comorbid neurodegenerative alterations, thereby imposing a severe healthcare burden on individuals, families, and society. Alzheimer’s disease (AD) and Parkinson’s disease (PD) represent the most common neurodegenerative pathologies, characterized by neuronal structural changes and progressive cognitive decline. An epidemiological study based on national demographic data found a significantly elevated incidence of AD among individuals with insomnia, with a poorer prognosis compared to those without non-insomnia (Baek et al., 2021). Further, a retrospective longitudinal cohort study in Taiwan demonstrated an increased risk of PD associated with insomnia (Hsiao et al., 2017).

Previous investigations have revealed that insomnia are commonly observed early in the progression of dementia, affecting approximately 44% of patients with AD, the highest incidence is found in those with AD and PD, where up to 90% of patients are affected (Ju et al., 2013). Insomnia can lead to various alterations in brain structure and function, including inflammation, degeneration, and changes in glial function. Consequently, they represent a risk factor for dementia and can worsen morbidity in dementia patients (Pillai and Leverenz, 2017; Gottesman et al., 2024). Conversely, alterations in brain structures and functions that are critical for sleep regulation can result in comorbid insomnia in dementia patients, indicating a bidirectional relationship between insomnia and changes in brain structure and function (Mutti et al., 2021). Previous cross-sectional studies have demonstrated that insomnia adversely affects cognitive function, particularly attention and memory, in patients with PD (Jozwiak et al., 2017). Insomnia may be a significant risk factor for cognitive impairment in PD patients. A longitudinal cohort study found that, among PD patients with insomnia, the annual decline in MoCA scores was higher over a 3-year follow-up period (Chahine et al., 2016). Presently, the reasons for the increased comorbidity risk between insomnia and AD/PD remain unclear, we speculate that changes in serum metabolites may be an important contributing factor to the common etiology between insomnia and AD/PD. Serum metabolic characteristics play a crucial role in the pathological processes of insomnia, AD, and PD. A significant increase in the levels of acylcarnitine, glycerophospholipids, and sphingolipids have been reported during acute sleep deprivation as compared to that during the sleep period (Davies et al., 2014). Besides, in comparison with healthy individuals, patients with insomnia exhibit significant alterations in phospholipid metabolites such as 1-palmitoylglycerophosphocholine (Wang et al., 2022). Phospholipids are essential components of biological membranes and play a crucial role in maintaining the structure and function of nerve cells. Untargeted metabolomic studies have revealed metabolic disruptions in carbohydrates, amino acids, and lipids in patients with AD compared to cognitively normal groups (Yu et al., 2023). In patients with PD, significant alterations occur in the levels of acylcarnitine, indolelactic acid, and unsaturated fatty acids, indicating a potential association between these metabolic characteristics and PD pathogenesis (Shao et al., 2021). While these observational studies confirm the correlation between serum metabolic characteristics and insomnia, AD, and PD, in this study, we aimed to explore causality through Mendelian randomization (MR), acknowledging the limitations of observational studies in elucidating causal relationships and accounting for confounding factors and reverse causation.

Previous studies have indicated associations between serum metabolites and various neurodegenerative diseases. However, these findings must be interpreted cautiously due to the potential for confounding factors and reverse causation. Unlike traditional observational studies that are prone to such biases, Mendelian randomization (MR) uses genetic variations as instrumental variables, offering a robust method to establish causal relationships. In this study, we employed MR to investigate the associations between serum metabolites and neuropsychiatric disorders such as insomnia, AD and PD. Through this analysis, we aim to contribute to a broader understanding of the underlying causes for the increased comorbidity risk between insomnia and AD/PD.

## 2 Materials and methods

### 2.1 Study design

We conducted a two-sample MR investigation to evaluate the causal relationships between 1,400 metabolic characteristics and the risk of insomnia, AD, widely defined AD (WDAD), and PD. The MR framework employs genetic variances as surrogates for risk factors, necessitating robust instruments that adhere to three fundamental assumptions crucial in causal inference: (1) direct association between genetic variances and the exposure, (2) absence of correlation between genetic variances and potential confounding factors bridging the exposure and outcome, and (3) no influence of genetic variances on the outcome via pathways distinct from the exposure (Lawlor, 2016).

### 2.2 Data sources

#### 2.2.1 Data sources for metabolic characteristics

We comprehensively analyzed a large-scale genome-wide association study (GWAS) and established instrumental variables (IVs) related to serum metabolic characteristics. The GWAS summary statistics for this study, with accession numbers GCST90199621-90201020 for European GWAS, have been deposited in the GWAS Catalog.^1^ This comprehensive GWAS delved into the intricate genetic foundations of 1,091 plasma metabolic characteristics and 309 corresponding ratios (Chen et al., 2023).

Of the 1,091 plasma metabolic characteristics, 850 presented unequivocal identities spanning eight super pathways, encompassing lipids, amino acids, xenobiotics, nucleotides, cofactors and vitamins, carbohydrates, peptides, and energy. The remaining 241 were either classified as unknown or only “partially” characterized. To unveil latent biological processes inherent in the study of individual metabolic characteristics, the researchers computed ratios at the metabolite level for pairs sharing an enzyme or transporter (Chen et al., 2023). This meticulous process leveraged associations between metabolic characteristics and proteins meticulously documented in the Human Metabolome Database.

In contrast to an uninformed methodology to derive metabolite ratios, their systematic approach, anchored in empirical evidence, preserves enhanced statistical power by judiciously testing a subset of metabolite ratios displaying heightened biological plausibility (Chen et al., 2023).

#### 2.2.2 Data sources for outcomes

The inclusion criteria for this analysis of GWAS data were confined to cohorts of European lineage. The GWAS summary statistics for insomnia were obtained from the FinnGen project (DATA FREEZE 9^2^). The FinnGen Project, a substantial genetic exploration initiative, focuses on elucidating the intricate interplay between genomic data and health attributes within Finnish and broader European populations. The R9 dataset encompasses an extensive reservoir of data, with a total sample size of 377,277 individuals (210,870 females and 166,407 males) actively participating in the investigative undertaking. A meticulous analysis was performed on an extraordinary 20,175,454 genetic variants, probing connections with a diverse array of disease endpoints (phenotypes), totaling 2,272. This substantial repository of genetic revelations was disseminated among collaborative affiliates, with the latest data release occurring in the initial quarter of 2022.

Acquiring GWAS data for insomnia, AD, WDAD, and PD involved the formal submission of a request to the investigators overseeing the FinnGen study for their approval. The dataset includes 13,393 individuals with WDAD, along with 36,384 control individuals. In addition, the dataset includes 9,301 patients with AD and 36,7,976 control individuals, 4,235 patients with PD and 37,3,042 control individuals, and also 4,235 patients with insomnia and 37,3,042 control individuals (Kurki et al., 2023).

### 2.3 Selection of IVs

In line with contemporary investigations (Sha et al., 2023; Yan et al., 2023), the significance level threshold for IVs related to each metabolite trait was set at 1 × 10^−5^. The pruning process in PLINK v1.90 was employed to remove single nucleotide polymorphisms (SNPs) (linkage disequilibrium r^2^ threshold <0.01 within a 10,000 kb span). The computation of F-statistics was performed for each IV to assess the robustness of instrumental strength and prevent the risks of weak instrumental bias. IVs for metabolite characteristics were retained for subsequent analyses, excluding those with suboptimal F-statistics (<10).

### 2.4 Statistical analysis

All computations were performed using R 4.3.1.^3^ To examine the causal relationship between 1,400 metabolic characteristics and outcomes, we employed advanced statistical techniques, including inverse-variance weighting (IVW) (Slob and Burgess, 2020), weighted median (Bowden et al., 2016), simple mode, and weighted mode via the MendelianRandomization package v0.4.3 (Yavorska and Burgess, 2017). Cochran’s Q statistic and corresponding *p* values were utilized to evaluate heterogeneity among the selected IVs (Greco et al., 2015). To address potential horizontal pleiotropy effects, the MR-Egger method was applied, which identifies horizontal multiplicity if the intercept term achieves significance (Bowden et al., 2015; Burgess and Thompson, 2017). Furthermore, the robust MR-PRESSO method was utilized to identify and exclude plausible horizontal pleiotropic outliers that could significantly influence estimation results within the MR-PRESSO package (Verbanck et al., 2018). Our analysis also incorporated visualizations such as scatter, funnel, and leave-one-out plots. Scatter plots confirmed the robustness of outcomes, demonstrating their insensitivity to outliers. Funnel plots graphically represented the resilience of correlation, emphasizing the absence of heterogeneity. Leave-one-out plots helped assess the impact of individual data points on overall analyses, systematically excluding each data point one at a time and revealing resultant changes; moreover, they facilitated a nuanced evaluation of the influence of individual observations on the stability and reliability of the statistical model.

## 3 Results

### 3.1 Preliminary MR analysis and selection of key metabolic characteristics

**Figure 1:**
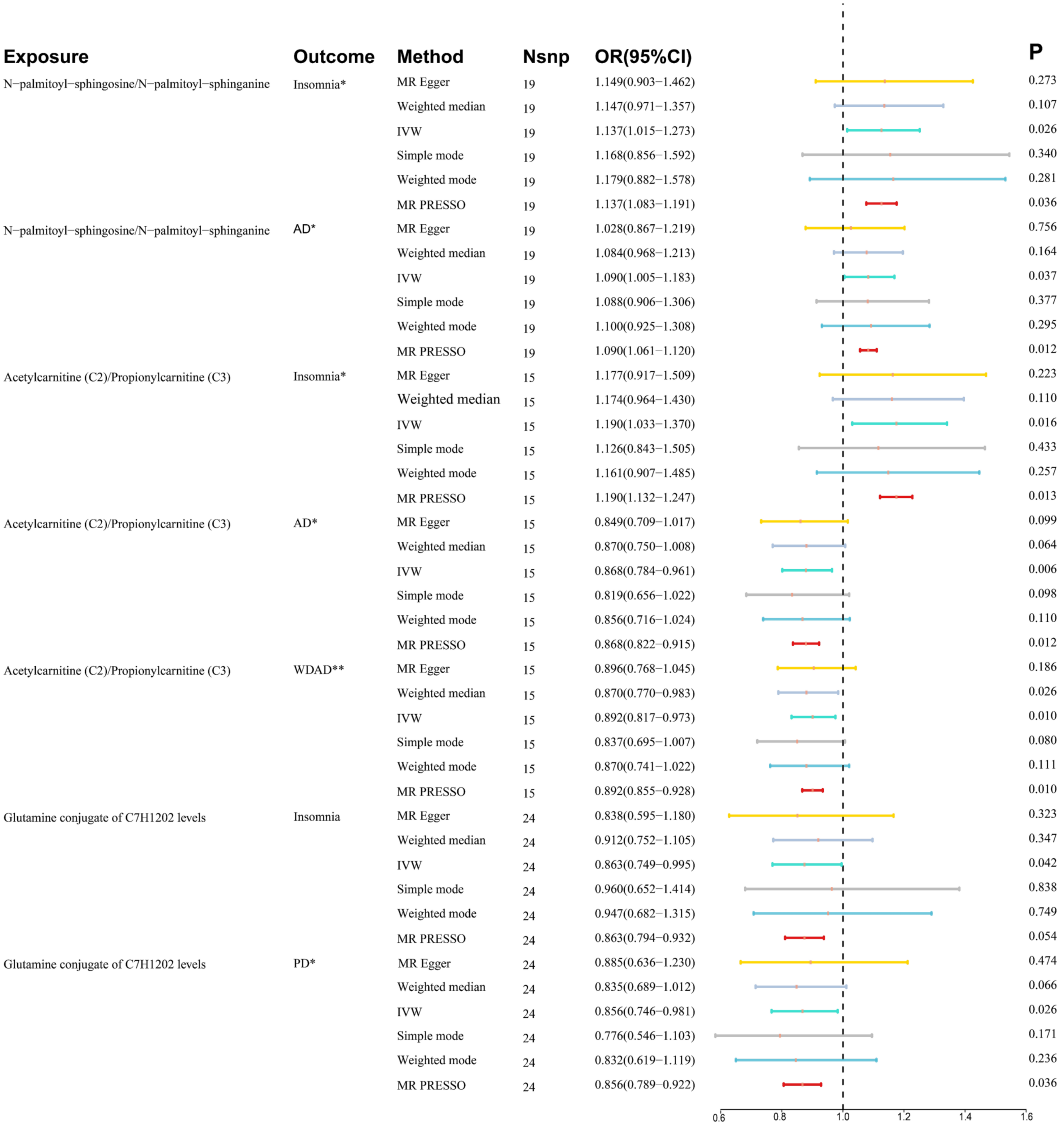
Summary of association between metabolic features and diseases. *Where P_IVW_ < 0.05 and P_weighted median_/P_MR-PRESSO_ < 0.05, it means suggestive association. **Where P_IVW_ < 0.05, P_weighted median_ and P_MR-PRESSO_ < 0.05, it means significant association. CI, confidence interval; IVW, inverse variance weighted; OR, odds ratio; Nsnp, number of single-nucleotide polymorphisms; AD, Alzheimer’s disease; WDAD, widely defined Alzheimer’s disease; PD, Parkinson’s disease.

**Figure 2:**
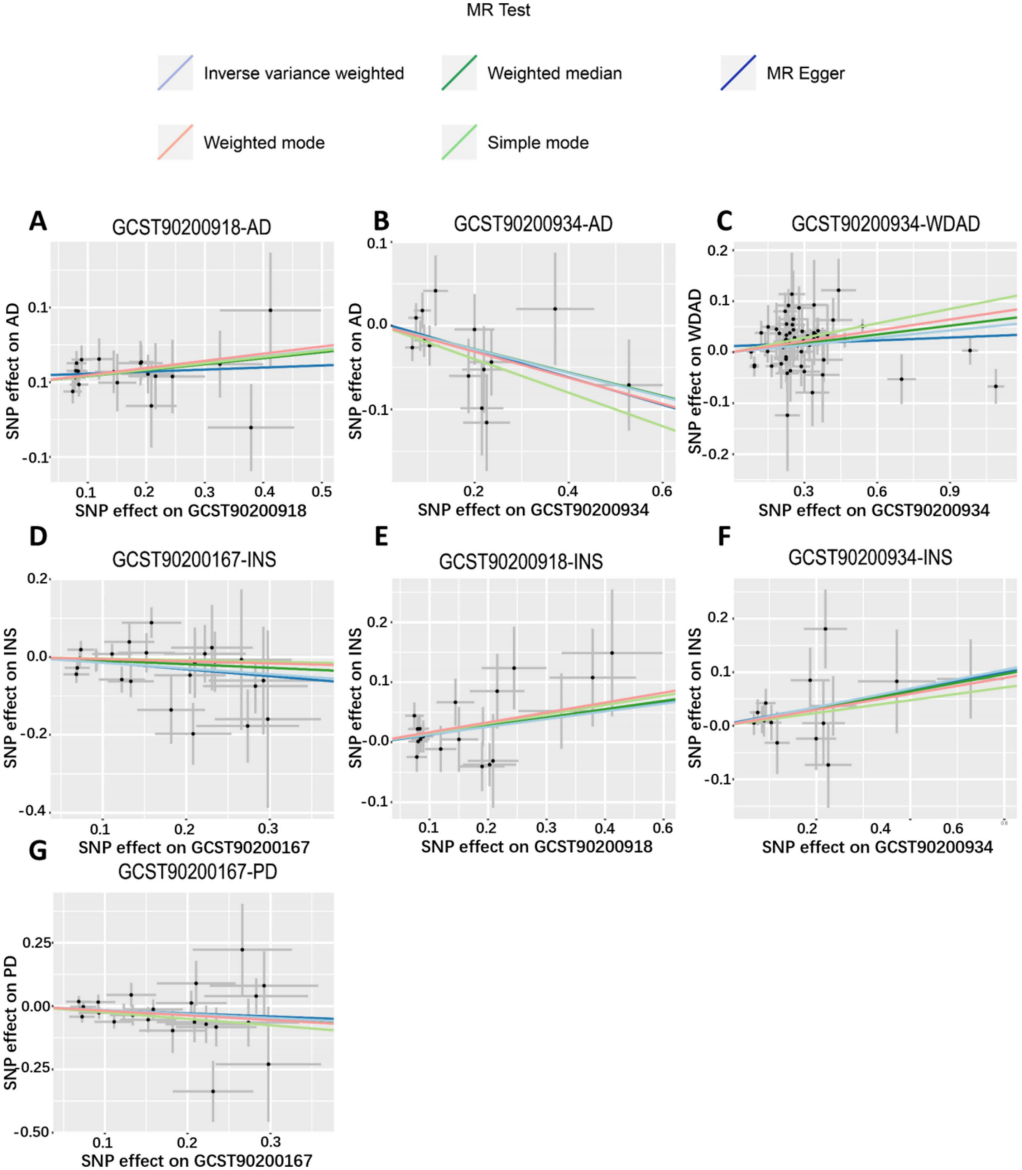
Scatter plots of genetic correlations of metabolic features and insomnia, WDAD, AD, and PD using different MR methods. **(A,E)** Scatter plot of genetic correlations of N-palmitoyl-sphingosine (d18:1 to 16:0) to N-palmitoyl-sphinganine (d18:0 to 16:0) ratio with AD and insomnia. **(B,C,F)** Scatter plot of genetic correlations of acetylcarnitine (C2) to propionylcarnitine (C3) ratio with AD, WDAD, and insomnia. **(D,G)** Scatter plot of genetic correlations of glutamine conjugate of C_7_H_12_O_2_ levels with insomnia and PD. AD, Alzheimer’s disease; WDAD, widely defined Alzheimer’s disease; PD, Parkinson’s disease.

**Figure 3:**
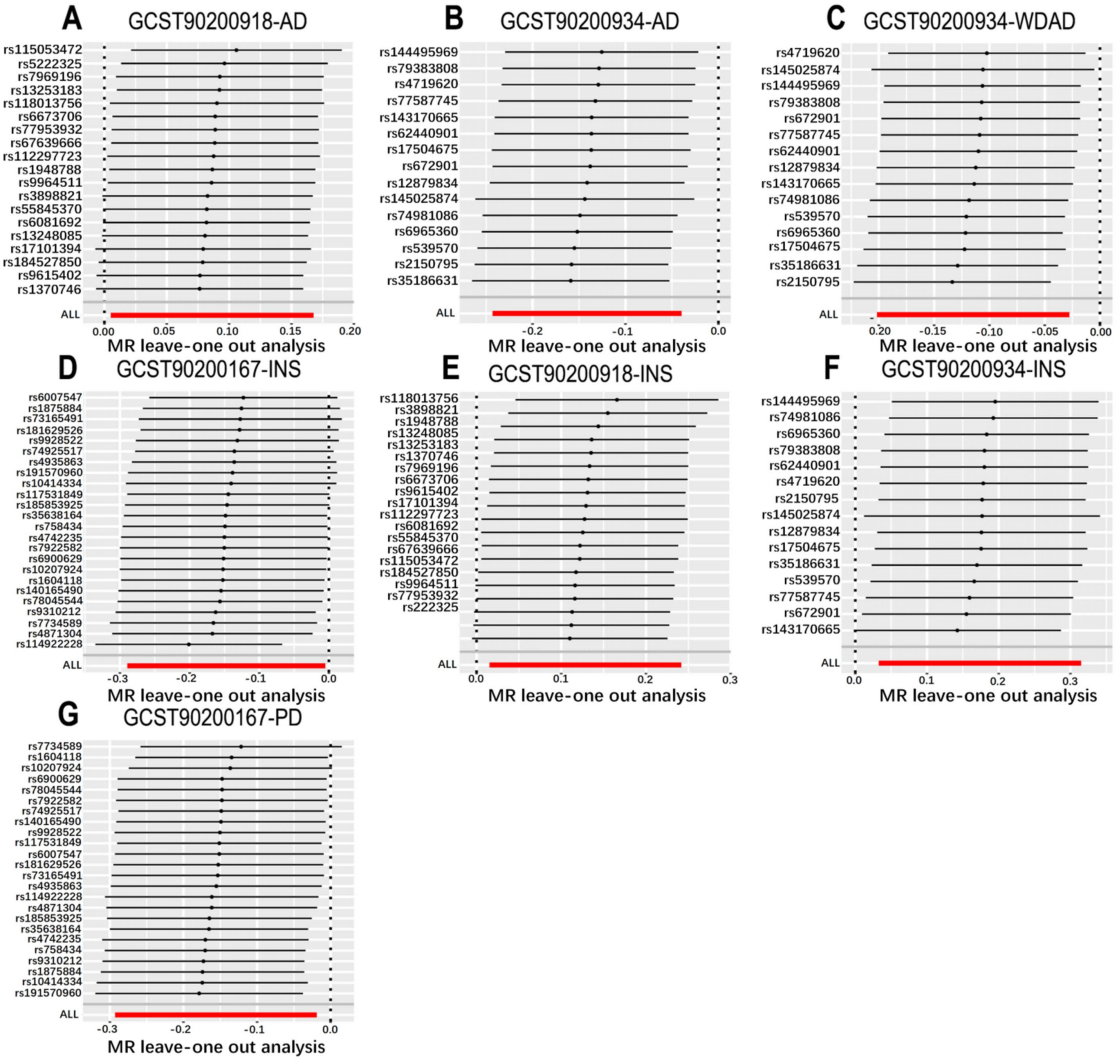
Leave-one-out plot from genetically predicted metabolic features on insomnia, WDAD, AD, and PD. **(A,E)** Leave-one-out plot of genetic correlations of N-palmitoyl-sphingosine (d18:1 to 16:0) to N-palmitoyl-sphinganine (d18:0 to 16:0) ratio with AD and insomnia. **(B,C,F)** The leave-one-out plot of genetic correlations of acetylcarnitine (C2) to propionylcarnitine (C3) ratio with AD, WDAD and insomnia. **(D,G)** Leave-one-out plot of genetic correlations of glutamine conjugate of C_7_H_12_O_2_ levels with insomnia and PD. AD, Alzheimer’s disease; WDAD, widely defined Alzheimer’s disease; PD, Parkinson’s disease.

**Figure 4:**
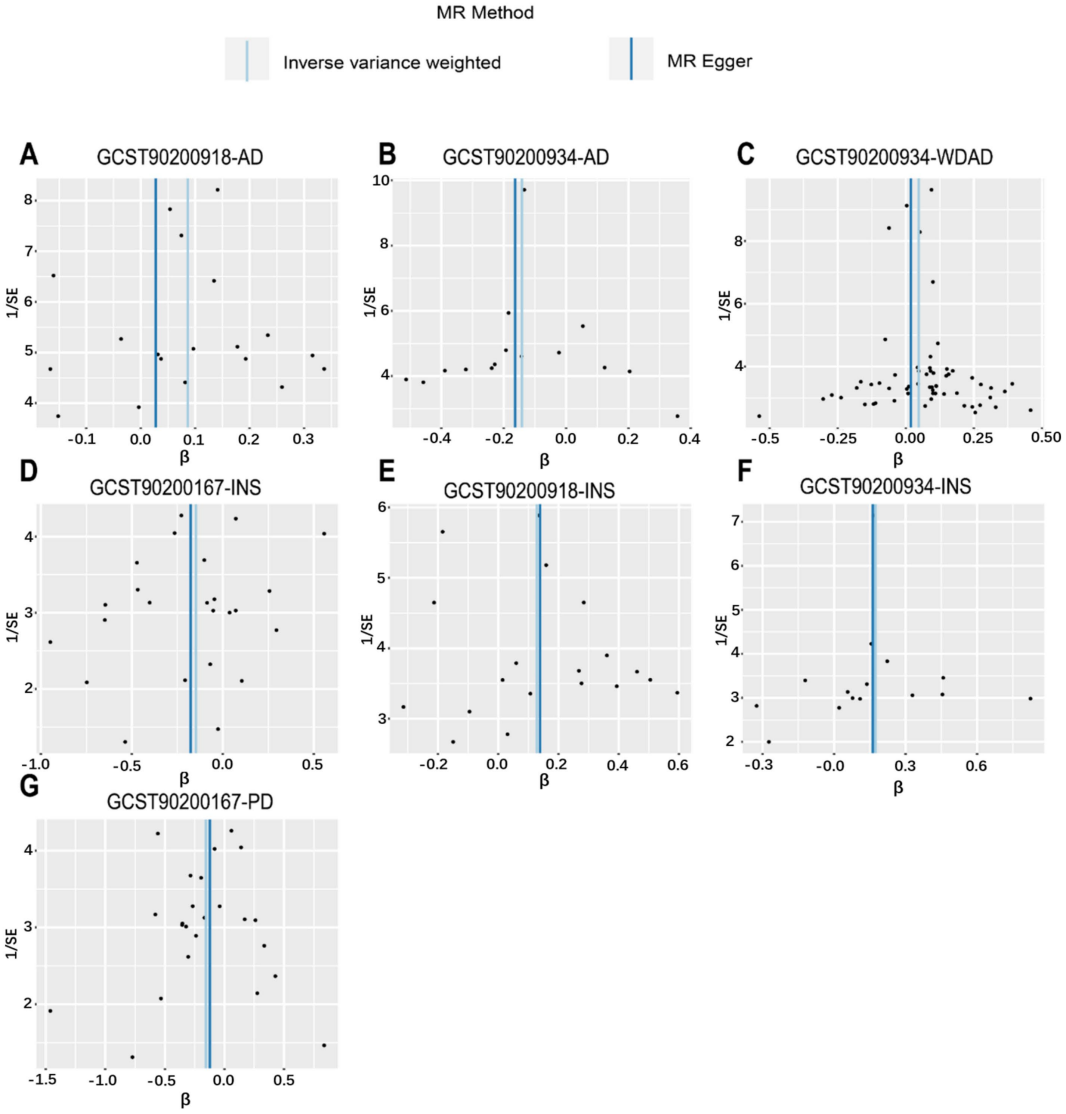
Funnel plot from genetically predicted metabolic features on insomnia, WDAD, AD, and PD. **(A,E)** Funnel plot of genetic correlations of N-palmitoyl-sphingosine (d18:1 to 16:0) to N-palmitoyl-sphinganine (d18:0 to 16:0) ratio with AD and insomnia. **(B,C,F)** Funnel plot of genetic correlations of acetylcarnitine (C2) to propionylcarnitine (C3) ratio with AD, WDAD, and insomnia. **(D,G)** Funnel plot of genetic correlations of glutamine conjugate of C_7_H_12_O_2_ levels with insomnia and PD. AD, Alzheimer’s disease; WDAD, widely defined Alzheimer’s disease; PD, Parkinson’s disease.

We performed MR analyses with 1,400 metabolic characteristics in relation to insomnia, AD, WDAD, and PD. Based on our preliminary findings, 40, 45, 50, and 50 metabolic characteristics were identified to be potentially causally related to insomnia (Supplementary Table S1), AD (Supplementary Table S2), WDAD (Supplementary Table S3), and PD (Supplementary Table S4), respectively. By curating frequently appearing metabolic characteristics and meticulous examining vertical and horizontal pleiotropy using IVW and the MR-Egger and MR-PRESSO methods (Table 1), we observed that N-palmitoyl-sphingosine/N-palmitoyl-sphinganine concurrently correlated causally with the onset risks of insomnia and AD, while acetylcarnitine (C2)/propionylcarnitine (C3) concurrently influenced the onset risks of insomnia, AD, and WDAD. Further, our findings suggested that N-acetyl-aspartyl-glutamate levels and glutamine conjugate of C_7_H_12_O_2_ levels concurrently influence the onset risks of insomnia and PD. With regard to positive outcomes, the total count of index SNPs chosen for the genetic prediction of metabolic characteristics (Supplementary Tables S5–S12) ranged from 15 to 59 (Figure 1). The F-statistics of these genetic instruments exceeded the commonly adopted threshold value of 10, affirming their robustness and potency (Supplementary Tables S5–S12). SNP effect sizes for the exposure were visualized via scatter plots for the outcome (Figures 2A–G). Leave-one-out sensitivity analysis indicated that no individual SNP significantly influenced the association between the exposure and outcome (Figures 3A–G). Finally, funnel plots revealed no significant heterogeneity among the chosen independent variables (Figures 4A–G).

**Table 1:** Results of heterogeneity and pleiotropy tests.

Exposure_ID	Exposure	Outcome	IVW Q_P	Egger intercept_P	MR-PRESSO Global Test_P
GCST90200918	N-palmitoyl-sphingosine to N-palmitoyl-sphinganine ratio	Insomnia	0.494	0.924	0.482
		AD	0.927	0.451	0.927
GCST90200934	Acetylcarnitine (C2) to propionylcarnitine (C3) ratio	Insomnia	0.765	0.916	0.799
		AD	0.579	0.776	0.655
		WDAD	0.728	0.940	0.752
GCST90200167	Glutamine conjugate of C_7_H_12_O_2_ levels	Insomnia	0.225	0.855	0.208
		PD	0.319	0.829	0.323

AD, Alzheimer’s disease; WDAD, widely defined Alzheimer’s disease; PD, Parkinson’s disease.

### 3.2 Causal association of N-palmitoyl-sphingosine/N-palmitoyl-sphinganine with insomnia and AD

The ratio of N-palmitoyl-sphingosine to N-palmitoyl-sphinganine was associated with increased risk of insomnia (MR IVW method: OR = 1.137, 95% CI = 1.015–1.273, *p* = 0.026) and AD (MR IVW method: OR = 1.090, 95% CI = 1.005–1.183, *p* = 0.037).

### 3.3 Causal association of acetylcarnitine/propionylcarnitine with insomnia, AD, and WDAD

The ratio of acetylcarnitine to propionylcarnitine emerged as a risk factor for insomnia (MR IVW method: OR = 1.190, 95% CI = 1.003–1.370, *p* = 0.016) while exhibiting a protective effect against AD (MR IVW method: OR = 0.868, 95% CI = 0.784–0.961, *p* = 0.006) and WDAD (MR IVW method: OR = 0.892, 95% CI = 0.817–0.973, *p* = 0.010). The consistency of the effects of the ratio of acetylcarnitine to propionylcarnitine on WDAD was validated in the subsequent sensitivity analyses (weighted median: OR = 0.870, 95% CI = 0.770–0.983, *p* = 0.026).

### 3.4 Causal association of glutamine conjugate of C7H12O2 levels with insomnia and PD

Glutamine conjugate of C_7_H_12_O_2_ levels were associated with decreased risk of insomnia (MR IVW method: OR = 0.863, 95% CI = 0.749–0.995, *p* = 0.042) and PD (MR IVW method: OR = 0.856, 95% CI = 0.746–0.981, p = 0.026).

## 4 Discussion

Previous observational studies have indicated an increased comorbidity risk of AD and PD in individuals with insomnia (Baek et al., 2021; Hsiao et al., 2017). Considering the limitations of observational studies and the significant role of serum metabolite-mediated oxidative stress reactions in patients with insomnia with comorbid AD and PD, herein we performed MR analyses to mitigate confounding factors and reverse causation. Our core objective was to elucidate the causal relationship between serum metabolic characteristics and insomnia, AD, and PD. We found that increased N-palmitoyl-sphingosine to N-palmitoyl-sphinganine ratio simultaneously increased the risk of insomnia and AD, while glutamine conjugate of C_7_H_12_O_2_ levels decreased the risk of insomnia and PD, indicative of shared etiology between insomnia and AD/PD. In addition, increased ratio of acetylcarnitine to propionylcarnitine was observed to be positively correlated with the risk of insomnia and negatively correlated with the risk of AD and ADWD.

There is a significant correlation between oxidative stress and insomnia. During wakefulness, neurons produce reactive oxygen species (ROS), while sleep facilitates the clearance of these ROS, thereby mitigating oxidative damage. Sleep deprivation leads to the accumulation of ROS and subsequent oxidative stress (Vaccaro et al., 2020). Our research reveals a causal relationship between insomnia and specific serum metabolite levels, including the N-palmitoyl sphingosine/dipalmitoyl sphingosine ratio, the acetylcarnitine/propionylcarnitine ratio, and the C7H12O2-glutamine conjugate. These metabolic alterations suggest that insomnia may not only be a symptom but also a potential pathophysiological contributor to neurodegenerative diseases such as AD and PD.

AD is the most prevalent clinical neurodegenerative disorder, characterized by the accumulation of abnormal proteins—*β*-amyloid plaques and tau tangles—within the brain, leading to neuronal dysfunction or death (Atlante and Valenti, 2023). Mitochondrial dysfunction and oxidative stress are critically implicated in the onset and progression of AD (Song et al., 2021). Mitochondrial impairment induces oxidative stress, reduces ATP synthesis, disrupts normal neuronal metabolism, and promotes the accumulation of *β*-amyloid and tau by enhancing β-secretase activity through elevated ROS levels. This ultimately affects the anatomy and physiology of neurons, resulting in cell death (Bell et al., 2021). We have observed that an elevated N-palmitoyl sphingosine/dipalmitoyl sphingosine ratio is associated with increased risk of insomnia and AD. Elevated levels of N-palmitoyl sphingosine may promote inflammation and apoptosis, potentially compromising the integrity of the blood–brain barrier (BBB) and exacerbating neurodegenerative processes (Huang et al., 2020). Similarly, an imbalance in the acetylcarnitine/propionylcarnitine ratio may indicate mitochondrial dysfunction, a critical factor in the development and progression of AD (Ke et al., 2021; McCann et al., 2021). Acetylcarnitine and propionylcarnitine are intermediates in fatty acid oxidation with potent antioxidant properties (Moskaleva et al., 2022). Their ratio can indirectly reflect oxidative stress levels (Solevåg et al., 2021), and alterations in this ratio may lead to increased deposition of abnormal proteins such as β-amyloid and tau (Islam, 2017), hallmark features of AD. Inhibiting the synthesis of these substances may offer new therapeutic strategies for neurodegenerative diseases like AD.

PD is the second most common neurodegenerative disorder after AD, characterized by a reduction in the number of dopaminergic neurons in the substantia nigra, leading to decreased dopamine levels in the basal ganglia (Korczowska-Łącka et al., 2023). Mitochondrial dysfunction and oxidative stress play pivotal roles in PD pathology. Research has shown that oxidative stress promotes the synthesis of cytotoxic compounds, which accumulate in dopaminergic neurons, leading to protein misfolding, enzyme inactivation, lipid peroxidation, and cell death. These processes ultimately contribute to the degenerative changes in dopaminergic neurons (Chakrabarti and Bisaglia, 2023; Dong-Chen et al., 2023). In this study, we found that elevated levels of the C7H12O2-glutamine conjugate are associated with a reduced risk of PD, suggesting that mitigating oxidative stress may be one of its underlying mechanisms. Glutamine, the most abundant free amino acid in the body, reduces oxidative stress by inhibiting the activation of the PI3K/Akt signaling pathway, thereby minimizing damage to dopaminergic neurons (Cruzat et al., 2018; Yan et al., 2020). This finding supports the hypothesis that reducing oxidative stress may serve as a protective mechanism in PD; however, further research is needed to determine whether the C7H12O2-glutamine conjugate exerts its antioxidant effects through this pathway.

The increased risk of comorbid insomnia with PD and AD imposes a significant healthcare burden on individuals, families, and society. However, our understanding of the shared pathophysiological mechanisms between insomnia and AD/PD remains incomplete. In this study, we employed serum metabolic profiling to identify risk and protective factors associated with insomnia, AD, and PD. Our findings confirm the existence of common pathophysiological mechanisms linking insomnia with AD/PD. We believe that serum metabolic profiling can aid in predicting neurodegenerative comorbidities in patients with insomnia, offering new avenues for the prevention and treatment of comorbid insomnia and AD/PD.

Although metabolites such as N-palmitoyl sphingosine and dipalmitoyl sphingosine have limited ability to cross the blood–brain barrier, thereby restricting their direct impact on insomnia and neurodegenerative diseases, changes in their levels in the peripheral system may reflect pathological alterations in the brain (Quinville et al., 2021). We propose that future research should investigate whether these metabolites are synthesized within the brain, as well as their local effects and potential connections between the brain and peripheral systems, to achieve a more comprehensive understanding of their roles in neurodegenerative diseases and insomnia.

This study has several merits. For example, while observational studies are limited to correlational analyses, MR studies facilitate causal inference, mitigating the impact of confounding factors and reverse causation. The inclusion of a large sample size enabled thorough MR analyses across various metabolic characteristics. Furthermore, we utilized the most recent genomic data for metabolic characteristics, which ensured comprehensive metabolite coverage. Open-access datasets and open-source software were employed, enhancing the transparency and reproducibility of our findings; moreover, rigorous testing for heterogeneity (Cochran’s Q) and pleiotropy (MR-PRESSO global and MR-Egger regression interval trial tests) was performed, demonstrating the reliability of our key results.

This study also has few limitations. While MR analysis serves as a robust tool to assess causal relationships between metabolic characteristics and diseases, validation through experimental data is imperative. Herein we only established the presence of shared etiology between insomnia and AD/PD, postulating that aberrations in metabolic characteristics potentially contribute to comorbidity. Further research needs to be conducted to explore specific pathophysiological mechanisms. It is notable that this study exclusively involved participants of European descent, potentially limiting the generalizability of our results to other ethnicities. We believe that future studies should employ a combination of observational and genetic methods to further explore the impact of serum metabolic characteristics on insomnia and AD/PD. This exploration should involve careful consideration of stratification factors, including age and gender.

## 5 Conclusion

To summarize, we performed MR analyses and identified several metabolic characteristics that were potentially causally related to insomnia, AD, WDAD, and PD. In particular, we identified a causal relationship between N-palmitoyl-sphingosine/N-palmitoyl-sphinganine and insomnia and AD and also that between acetylcarnitine/propionylcarnitine and insomnia, AD, and WDAD. Further, glutamine conjugate of C_7_H_12_O_2_ levels were causally associated with insomnia and PD. Our results provide novel insights into the pathological mechanisms underlying the comorbidity of insomnia and AD/PD and should contribute to the development of new therapeutic approaches. Further investigations are warranted to validate our findings and elucidate potential molecular mechanisms linking these metabolites to insomnia and neurodegenerative conditions (Abad et al., 2018).

## Data Availability

The original contributions presented in the study are included in the article/Supplementary material, further inquiries can be directed to the corresponding authors.

## Footnotes

^1^ https://www.ebi.ac.uk/gwas/

^2^https://www.finngen.fi/en, accessed on November 28, 2023.

^3^ http://www.Rproject.org
